# Clinical stakeholders’ opinions on the use of selective decontamination of the digestive tract in critically ill patients in intensive care units: an international Delphi study

**DOI:** 10.1186/cc13096

**Published:** 2013-11-08

**Authors:** Brian H Cuthbertson, Marion K Campbell, Graeme MacLennan, Eilidh M Duncan, Andrea P Marshall, Elisabeth C Wells, Maria E Prior, Laura Todd, Louise Rose, Ian M Seppelt, Geoff Bellingan, Jill J Francis

**Affiliations:** 1 Department of Critical Care Medicine, Sunnybrook Health Sciences Centre, 2075 Bayview Avenue, Toronto ON M4N 3 M5, Canada; 2 Department of Anesthesia, University of Toronto, Room 121, Fitzgerald Building, 150 College Street, Toronto, Ontario M5S 3E2, Canada; 3 Health Services Research Unit, Health Sciences Building, Foresterhill, University of Aberdeen, Aberdeen, UK; 4 Aberdeen Health Psychology Group, Health Services Research Unit, Health Sciences Building, Foresterhill, University of Aberdeen, Aberdeen, UK; 5 Faculty of Nursing, Gold Coast Hospital and Griffith Health Institute, Griffith University, Parklands Drive, Southport, Queensland, Australia; 6 Centre for the Study of Social and Legal Responses to Violence, University of Guelph, Guelph, Ontario, Canada; 7 Trillium Gift of Life Network, 522 University Avenue, Toronto, Canada; 8 Lawrence S. Bloomberg Faculty of Nursing, University of Toronto, 155 College Street, Toronto, Canada; 9 Sydney Medical School - Nepean, Kingswood, University of Sydney, Sydney, New South Wales, Australia; 10 Intensive Care Unit, University College Hospital, 235 Euston Road, London, UK

## Abstract

**Introduction:**

Selective decontamination of the digestive tract (SDD) is a prophylactic antibiotic regimen that is not widely used in practice. We aimed to describe the opinions of key ‘stakeholders’ about the validity of the existing evidence base, likely consequences of implementation, relative importance of their opinions in influencing overall practice, likely barriers to implementation and perceptions of the requirement for further research to inform the decision about whether to embark on a further large randomised controlled trial.

**Methods:**

This was a Delphi study informed by comprehensive framework of possible determinants of health professionals’ behaviour to study Critical Care practice in four countries. There were four key stakeholder participant groups including ICU physicians, pharmacists, clinical leads, and clinical microbiologists/ infectious disease physicians. Round one comprised participant interviews and Rounds two and three were online questionnaires using Delphi method.

**Results:**

In this study, 141 participants were recruited of whom 82% were retained. Participants rated themselves as knowledgeable about SDD. Antibiotic resistance was identified as the most important issue. SDD was seen as a low clinical priority but few participants reported strong opposition. There was moderate agreement that research to date has not adequately addressed concerns about antibiotic resistance and lacks generalizability. Participants indicated equipoise with regard to benefits and harms of SDD, and indicated strong support for a further randomised trial.

**Conclusions:**

Clinicians have clinical equipoise about the effectiveness of SDD. Future research requires longer follow up to assess antibiotic resistance as well as greater validity/generalizability to provide definitive answers on the effectiveness of decontamination and effects on antibiotic resistance. SDD was regarded as not being a high clinical priority, which may limit future trial participation. These results have identified that further large randomised controlled trial of SDD in critical care is both warranted and appropriate.

## Introduction

Hospital-acquired infections (HAI) are a major clinical problem for modern health services because they are associated with morbidity and mortality as well as high additional healthcare costs. Critically ill patients are extremely susceptible to such infections. Between 20% and 50% of ICU patients experience HAI [1]. Reducing the incidence and mortality of HAI is currently the focus of many ICU quality improvement programmes and government initiatives worldwide.

One intervention that has gained much attention in reducing the incidence of HAI is selective decontamination of the digestive tract (SDD). SDD involves the application of topical nonabsorbable antibiotics to the oropharynx and stomach, and a short course of intravenous antibiotics [2-9]. The evidence supporting the use of SDD currently comprises 12 meta-analyses of 36 randomised controlled trials (RCTs) [2-9]. Many of these studies demonstrate a benefit in terms of reducing rates of pneumonia, and more recent studies also show lower mortality in all ICU patients or in certain subgroups. A recent large cluster RCT of SDD conducted in the Netherlands found a 3.5% absolute reduction in mortality [10]. A Cochrane review of SDD comprising 36 trials demonstrated that SDD was associated with reduced pneumonia (odds ratio 0.32 (95% confidence interval 0.26 to 0.38)) and death (odds ratio 0.75 (95% confidence interval 0.65 to 0.87)) [9]. This mortality benefit is of the magnitude of a 3 to 6% absolute risk reduction with a number needed to treat of 18 to save one life [9,10]. If the documented mortality benefit could be realised internationally it would save tens of thousands of lives each year.

Despite this evidence, the international ICU community has not widely adopted SDD [11]. Little systematic evidence is available about clinicians’ beliefs regarding the existing evidence, the perceived benefits and risks of SDD, the factors that influence lack of adoption, and likely barriers to implementation [11]. Further, it is unclear whether there is a need for further evidence of effectiveness before use of SDD would become broadly acceptable, and which study design would be feasible and acceptable to clinicians.

We performed an international Delphi study to identify the opinions of key stakeholders about the strength and generalisability of the existing evidence related to SDD, the positive and negative consequences of implementing SDD in ICUs, and the barriers to implementing SDD in ICUs. Ultimately, we planned to use the results of this Delphi study to inform practitioners and researchers about whether a further RCT of SDD in critical care was appropriate and warranted. To ensure the results generated would be maximally robust and systematic, we used the Theoretical Domains Framework of clinical behaviour developed from the field of health psychology [12,13]. The Framework provides a model for comprehensive assessment of factors affecting clinical behaviour.

## Materials and methods

An international Delphi study (one qualitative interview round and two quantitative rounds) was performed in participants in Australia and New Zealand, Canada, and the UK. Ethics approval was granted by the Nepean Research Ethics Committee (Australia and New Zealand), the Sunnybrook Hospital Research Ethics Committee (Canada) and the North of Scotland Ethics Service (the UK), and individual consent was obtained from all participants. All human and animal studies have been approved by the appropriate ethics committee and have therefore been performed in accordance with the ethical standards laid down in the 1964 Declaration of Helsinki and its later amendments.

### Sample

A Delphi process gauges opinions from a panel of experts [14]. For this study, we defined experts as those in a position to exert decisional authority and be key stakeholders with regard to ICU policy about SDD or to lead its implementation into clinical practice (if that was to happen). Experts were not defined as experts in SDD. We identified four key stakeholder groups: ICU physicians, ICU pharmacists, ICU clinical leads (medical and nursing), and clinical microbiologists and infectious disease specialists who had ICU responsibility.

Our target sample size was 10 participants per stakeholder group in each of the three geographical zones (40 per zone, 120 in total) [15]. The research team compiled lists of their respective stakeholder groups and assessed individuals according to predetermined diversity factors (geographical location, ICU bed number, current SDD practice and academic affiliation). To achieve a final sample size of 10 in all stakeholder groups in each zone, we initially oversampled up to three participants in each of the four groups per zone. Round 1 was a telephone-delivered semi-structured interviewing using a topic guide developed using the Theoretical Domains Framework. All participants who participated in Round 1 were invited to participate in Round 2. Only those who responded at Round 2 were invited to participate in Round 3. Full analysis of the Round 1 interviews of this Delphi study are reported elsewhere due to the differing nature of the data (qualitative), and the high total volume of data from the study makes concise reporting of all data in one paper impossible [16].

### Development of the Delphi materials

#### Use of the Theoretical Domains Framework

Construction of the Delphi materials was directly informed by the Framework, a comprehensive characterisation of possible determinants of health professionals’ behaviour. The Framework was developed out of a collaboration of health psychologists and implementation researchers, and has been used in other clinical spheres to systematically assess barriers and facilitators to the uptake of specific clinical behaviours [12]. The framework clusters determinants of health professionals’ behaviour into 12 salient domains (Table 1). The domains explore the effect of issues including knowledge (that is, how a clinician’s knowledge about a topic affects his/her behaviour), social/professional role and identity (how the accepted clinical thinking and norms of a particular profession affect a clinician’s behaviour), and beliefs about consequences (that is, how a clinician’s perception of the benefits and harms of a clinical action affects his/her behaviour).

**Table 1:** Explanations of the twelve theoretical domains used to generate Round 1 data

**Domain label **[12]	**Domain content**
Beliefs about consequences	Often regarded as core to clinical reasoning, this domain covers the perceived benefits and harms of a clinical action. In some contexts it can also include consequences for the clinician such as workload, pay, career progression, or for the hospital or health service.
Behavioural regulation	Includes the ‘how’ of changing clinical practice: what are the practical strategies that would facilitate or hinder uptake of a new practice.
Beliefs about capabilities	How confident clinicians are that they could change their practice effectively.
Emotion	Includes issues such as work stress, patient anxiety and other emotional factors that may help or hinder the uptake of new approaches to care.
Environmental context/resources	Includes the physical (including financial) issues that may limit change, including staffing levels and time as well as equipment or space.
Knowledge	Knowledge of the field (that is, whether there is adequate evidence) and individuals’ knowledge of the evidence or of a guideline.
Memory, attention and decision processes	The level of attention that is needed to perform the key clinical action (that is, whether forgetting is likely to be a problem) and the processes by which clinical decisions are made by individuals and teams.
Motivation and goals	The relative priority that is given to one clinical issue, compared with other demands.
Social/professional role and identity	The clinical thinking and norms of a particular profession.
Skills	Covers the possibility that new skills would be required by the staff that are required to implement a new procedure.
Social influences	The influence of other individuals or groups on clinical practice; for example, patients, patients’ families, pressure groups.
Nature of the behaviours	Some new practices are very similar to current practice and so are easier to implement than new practices that require a dramatic change in ways of working.

An added benefit of the Theoretical Domains Framework is that future research, if any, is guided by the theoretical domains identified as most crucial to the use of SDD. For example, if the beliefs about consequences domain was identified as most important, this would suggest that there are continuing uncertainties about the evidence base and that further research on clarifying the evidence base is required.

We ensured that all opinions raised in all regions in Round 1 were taken forward to Round 2 as items. Fifty-six items were generated in Round 1, 46 of which were common across all regions [16] and were included in Round 2 and Round 3 materials for the entire sample. In addition, five items with multiple response options were included in Rounds 2 and 3 to explore specific trial design and feasibility issues.

#### The Delphi rounds

Round 1 of the Delphi study involved semi-structured Theoretical Domains Framework-based interviews, analysed using content analysis. The set of 56 statements summarising beliefs about SDD generated from interview data were included in the instrument used for Round 2 and Round 3 reported in this paper. Item wording (that is, in favour of/against SDD) was selected as follows: using participants’ wording from Round 1 interviews; creating balance (positive and negative statement directions) among items; and reflecting best practice in terms of constructing questionnaire items. This resulted in eight positively worded items (that is, in favour of SDD use), 33 negatively worded items (that is, concerns about SDD use) and 11 neutrally worded items. For each item, there were two questions: ‘To what extent do you agree or disagree?’ (on a nine-point Likert scale, with 1 = ‘strongly disagree’ and 9 = ‘strongly agree’) and ‘How important is this issue is in your overall opinion about the delivery of SDD to critically ill patients?’ [17]. Five further questions were included to measure views about further SDD research (Additional file 1).

The Round 2 questionnaire was piloted with five clinicians not participating in the Delphi study and led to minor changes only. In Round 3 the same questionnaire was used together with feedback about the participant cohort responses to each question, a reminder of their own previous response and a request to rate the item again. Emails were sent to all Round 1 participants with individual links to the online questionnaire. Responses were monitored and reminders sent on two occasions within the month.

With regard to consensus, in this study we were interested not only in the proportion of participants who agreed with each item, but also the proportion of participants who were uncertain about their agreement with the items. Levels of consensus for the question ‘To what extent do you agree or disagree’ were assessed by noting the highest percentage of participants whose scores fell within any three-point band on the nine-point scale [18].

#### Data management and analysis

Descriptive statistics were calculated for participant demographics and for each statement, including the mean, median and interquartile range (IQR). We measured stability of opinions across rounds, both at individual participant and group response levels. At the group level, stability for each item was assessed by computing the change in arithmetic means across the whole sample from Rounds 2 and 3. A change of one point in the mean agreement level was deemed a potentially important change in opinion. At the individual level, stability was measured using individual change scores, such that a score of zero signified identical responses in Rounds 2 and 3.

## Results

### Participants

We recruited 141 participants into Round 1 and retained 118 to the end of Round 3 (82% retention) [16]. The breakdown of participation by stakeholder group and retention in the study is displayed in Additional file 2. Median years in practice was 15 (IQR 11 to 21) and median number of ICU beds was 20 (IQR 15 to 28), with 109 participants working in academic/academic-affiliated centres and nine in nonacademic centres.

### Participants’ self-reported knowledge of SDD

Participants’ perception of their own knowledge was measured with the item ‘I know the SDD evidence base well enough to have an informed opinion of its use’. Overall, participants rated themselves as knowledgeable about SDD (median score ≥6). However, 37 (31%) rated themselves <5 on the nine-point scale. Participants reporting a low level of perceived knowledge in Round 2 showed greater change in their responses from Round 2 to Round 3 (mean change 0.19, standard deviation 0.19) than participants whose perceived knowledge was >5 (mean change 0.09, standard deviation 0.13; *P* = 0.002).

### Stability of opinions

There was high stability of responses from Round 2 to Round 3, both at the individual level (>69% of change scores in the range −1 to +1) and at the group level (mean differences ranging from 0 to 0.52). Stability across stakeholder groups showed there was a greater level of change from Round 2 to Round 3 in the clinical lead group (mean 0.18, standard deviation 0.2) than in other stakeholder groups (means 0.09 to 0.11, standard deviation 0.16 to 0.2; *P* = 0.044).

### What was important to responders?

Questions rated as most important by participants are presented in Table 2. Two questions on antibiotic resistance were in the top three for importance. The validity of self-reported importance is a key issue as the ratings represent participant’s opinions and could be thought of as low level of evidence. However, in support of validity, we found a high level of congruence between these importance ratings and other indicators of importance [19].

**Table 2:** Most important items after Round 3 of the Delphi

**Domain of TDF **[12]	**Item stem**	**Median (IQR)**
Beliefs about consequences	SDD increases antibiotic resistance	8 (7 to 9)
Decision processes	The decision to adopt SDD requires consensus between my colleagues	8 (7 to 9)
Knowledge	Research to date has not adequately addressed concerns about antibiotic resistance and SDD	8 (7 to 9)
Decision processes	The decision to adopt SDD requires a review and appraisal of the current best evidence	8 (7 to 9)
Behavioural regulation	My hospital tries to reduce antibiotic use	8 (7 to 9)
Decision processes	Part of the decision to adopt SDD requires agreement about which patients will receive it	8 (7 to 9)
Beliefs about consequences	SDD would increase ICU *Clostridium difficile* infections	8 (6 to 8)
Knowledge	I know the SDD evidence base well enough to have an informed opinion regarding its use	8 (6 to 8)
Motivation	We are addressing hospital-acquired infections using other strategies	7 (6.5 to 9)
Motivation	We are addressing ventilator-associated pneumonia using other strategies	7 (7 to 8)

Data presented as medians and interquartile ranges on scale of 1 to 9 (1 = ‘strongly disagree’ and 9 = ‘strongly agree’). IQR, interquartile range; SDD, selective decontamination of the digestive tract; TDF, Theoretical Domains Framework.

### Opinions about SDD

**Figure 1:**
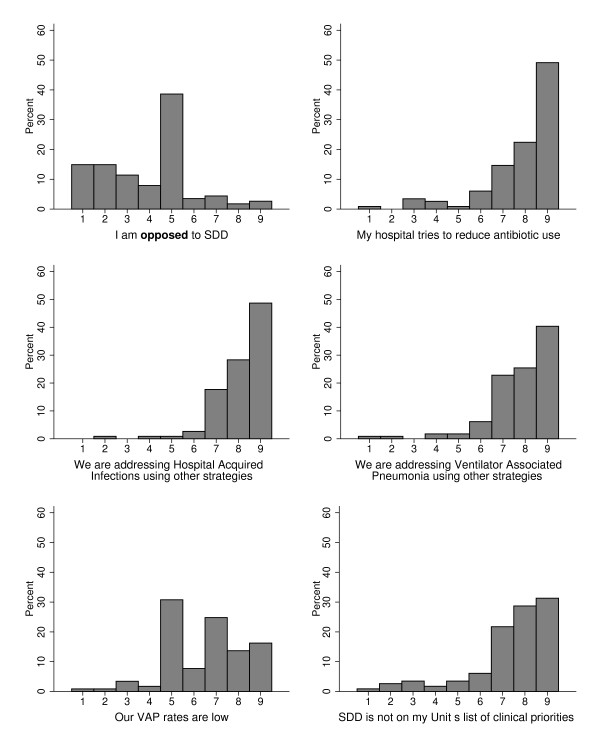
**Opinions about the relevance of selective decontamination of the digestive tract.** Response format: 1 = ‘strongly disagree’ to 9 = ‘strongly agree’. *y* axis, percentage of responders. SDD, selective decontamination of the digestive tract; VAP, ventilator-associated pneumonia.

Very few participants reported strong opposition to SDD (although scores ranged from 1 to 9; Figure 1). Participants indicated that their hospitals tried to reduce antibiotic use, reduce the widespread use of other strategies for tackling HAI, had low ventilator-associated pneumonia (VAP) rates and the lack of clinical priority for SDD (Figure 1). More items related to this topic are reported in Additional file 1.

### Opinions on the validity and adequacy of the evidence base and the consequences of implementing SDD

**Figure 2:**
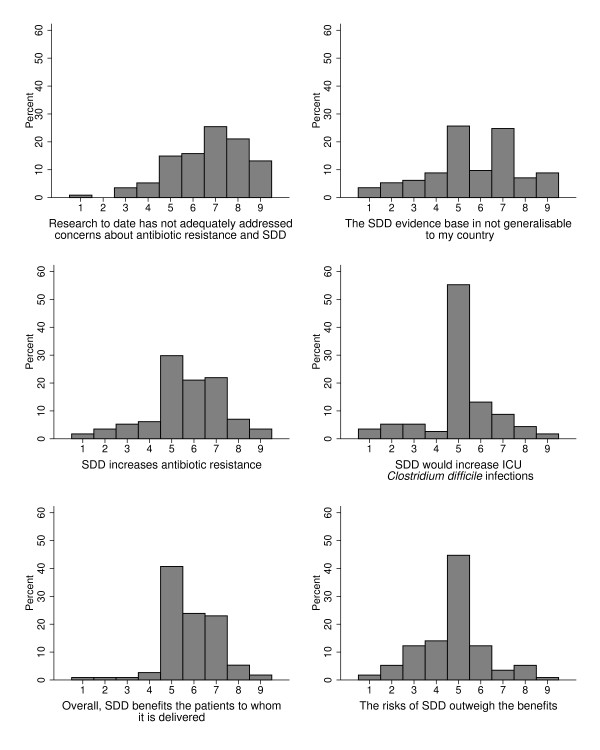
**Opinions on the internal and external validity and adequacy of the existing evidence base for selective decontamination of the digestive tract (SDD) and the likely consequences of implementing SDD in ICUs.** Top two graphs: opinions on the internal and external validity and adequacy of the existing evidence base for selective decontamination of the digestive tract (SDD). Bottom four graphs: opinions on the likely consequences of implementing SDD in ICUs. Response format: 1 = ‘strongly disagree’ to 9 = ‘strongly agree’. *y* axis, percentage of responders.

There was moderately strong agreement (median 7, IQR 6 to 8) that research to date has not adequately addressed concerns about antibiotic resistance, and that the evidence base is not generalisable (Figure 2). There was low-level agreement (median 6, IQR 5 to 7) that SDD increases antibiotic resistance but neutrality about whether SDD increases *Clostridium difficile*, whether SDD benefits the patients to whom it is delivered and whether the risks of SDD outweigh the benefits. More items related to these topics are reported in Additional file 1. Generally, these responses suggest clinical equipoise relating to the benefits and harms of SDD.

### Opinions about the likely barriers to implementation

**Figure 3:**
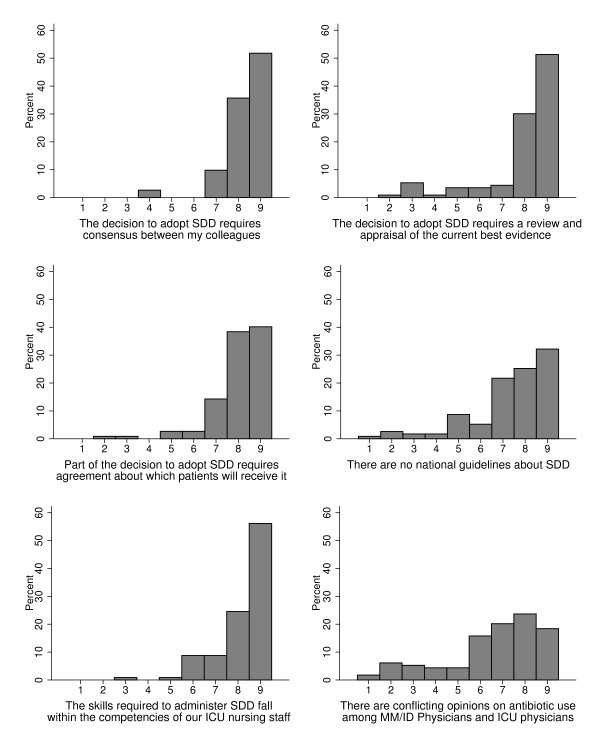
**Opinions about the likely barriers to implementing selective decontamination of the digestive tract in ICUs.** Response format: 1 = ‘strongly disagree’ to 9 = ‘strongly agree’. *y* axis, percentage of responders. MM/ID, microbiologist/infectious disease specialists; SDD, selective decontamination of the digestive tract.

There was strong agreement that the decision to adopt SDD requires consensus amongst colleagues (median 9, IQR 8 to 9), appraisal of the current evidence base (median 9, IQR 8 to 9), and agreement on which patients will receive SDD (median 8, IQR 8 to 9). There was strong agreement (median 9, IQR 8 to 9), that the skills to administer SDD fall within existing clinical competencies (Figure 3). Participants reported their perceptions of conflicting opinions on antibiotic use among microbiology and intensive care physicians (Additional files 3 and 4).

### Opinions on feasibility of further research

**Figure 4:**
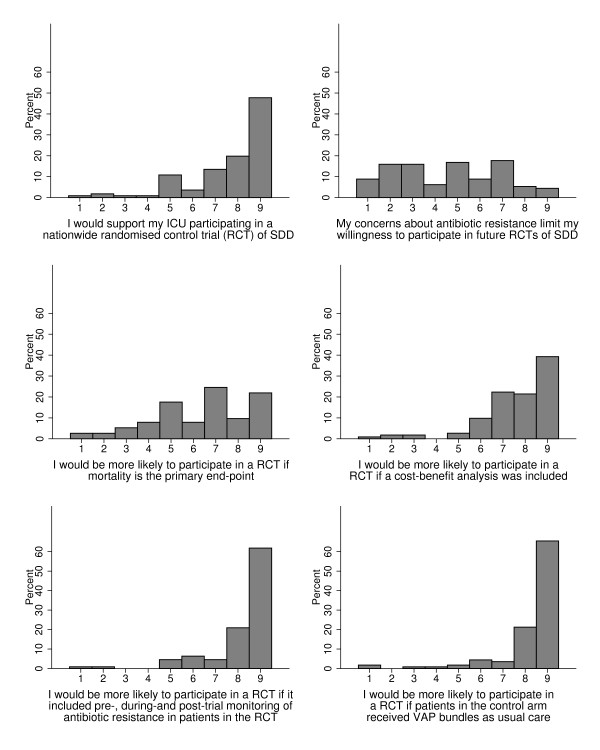
**Opinions on the feasibility of further selective decontamination of the digestive tract research and whether professional groups are likely to participate.** Response format: 1 = ‘strongly disagree’ to 9 = ‘strongly agree’. *y* axis, percentage of responders. SDD, selective decontamination of the digestive tract; VAP, ventilator-associated pneumonia.

There was strong agreement from participants regarding the need for an international RCT of SDD but there was significant variability with regard to the impact of concerns about antibiotic resistance on willingness to participate (Figure 4). Participants felt it was ethically acceptable to conduct further RCTs evaluating the effectiveness of SDD (median 8, IQR 7 to 9). Participants were in favour of mortality as a primary outcome, a cost–benefit analysis, monitoring of antibiotic resistance before, during and after the trial, and a control arm that would include either VAP bundles (the UK and Canada) or usual care (Australia/New Zealand) (Figure 4). More items related to this topic are reported in Additional file 1.

### Comparisons between stakeholder groups

We compared responses from the four different stakeholders groups for the items ‘I am opposed to SDD’ and ‘The risks of SDD outweigh the benefits’. For ‘I am opposed to SDD’, ICU physicians had a median 5 (IQR 3 to 5), microbiologists a median 6 (IQR 5 to 7), ICU pharmacists a median 5 (IQR 4 to 5) and clinical leads a median 5 (IQR 3 to 5). For ‘The risks of SDD outweigh the benefits’, ICU physicians had a median 3 (IQR 2 to 5), microbiologists a median 5 (IQR 4 to 7), ICU pharmacists a median 5 (IQR 3 to 6) and clinical leads a median 3 (IQR 2 to 5). Figures are presented in Additional files 3 and 4.

## Discussion

This is the first study to rigorously examine barriers to implementation of SDD and the first to examine these barriers with an international perspective. We also believe that using the Delphi technique to inform the decision to undertake a further large RCT is novel.

### Opinions about SDD

We did not find significant opposition to SDD despite agreement that the evidence base is currently seen as unconvincing and the fact that very few centres have implemented SDD. Participants identified that their hospitals were currently targeting VAP and HAI using other methods, and SDD was not a topic of clinical discussion. This perceived low priority of SDD could act as a major challenge to SDD implementation or to the performance of a future trial. When we analysed differences between key stakeholder groups we found that microbiologists were more likely to be opposed to SDD and, along with ICU pharmacists, were more likely to think that ‘the risks of SDD outweigh the benefits’. This could be explained by the microbiologists and the ICU pharmacists having a more ecological view on antibiotic usage whereas ICU clinicians may be more able to identify with the individual patient benefits.

### Opinions about the evidence base

Participants were not persuaded of the internal or external validity of the evidence base and demonstrated clinical equipoise with regard to the benefits and harms of SDD. We conducted a detailed examination of responses in the UK data to check whether equipoise was associated with poor knowledge of the evidence base, and found that the mid-point on the beliefs about consequences items was endorsed by a large proportion of participants (*n* = 21) who assessed their knowledge of the evidence base as high. Hence, equipoise was unlikely to have arisen from ignorance of the evidence base. Superficially, this equipoise would seem surprising in the presence of such a large evidence base suggesting mortality benefit [9], but since the literature to date does not clearly identify the effects of SDD on key issues such as antibiotic resistance, this seems a reasonable stance and is a key external validity issue. That said, evidence to date does not suggest harmful effects of SDD on antibiotic resistance [20]. The findings about equipoise have clear implications for further research. If there is certainty about the benefits of a clinical intervention, it is regarded as unethical to conduct further evaluation by randomising patients to a usual care control group [21]. Consistent with this principle, there was a high level of agreement that it would be ethically acceptable to conduct further SDD research.

It is interesting to compare the evidence base for SDD (which has not been widely implemented) with the evidence for low-dose steroids [22] or tight glycaemic control [23]. SDD remains not widely implemented despite a large (although clearly incomplete) evidence base, whereas low-dose steroids in septic shock and tight glycaemic control were implemented extraordinarily widely with only one supportive RCT and, in the case of steroids, previous evidence of harm [24]. This possibly suggests that extra-scientific factors are at play in such examples.

### Barriers to implementation

This finding of clinical equipoise was confirmed when we identified that issues related to benefits and harms of SDD were also the most important barriers to implementation. The decision process (to adopt) and consensus development were also identified as barriers. The need for appraisal of evidence was also identified as a barrier, which seems surprising considering the high number of meta-analyses in the literature. These issues may explain the very low levels of implementation to date, with no ICUs in Canada, Australia and New Zealand currently undertaking SDD and only 5% of ICUs in the UK undertaking SDD (RR Canter, S Harvey, DA Harrison, *et al*., Survey and observational analysis of current use of selective decontamination of the digestive tract in UK critical care units, *British Journal of Anaesthesia* 2013, submitted). Other practice issues such as ease of delivery, skills, patient side-effects and cost were not identified as barriers. Nursing and pharmacy workloads were identified as likely to increase but were not identified as barriers as such. Few participants delivered SDD in their practice and were therefore not ideally placed to make these assessments. Interviews with clinical staff in centres delivering SDD provided a similar perspective [25]. These results indicate the need for new research to address the ongoing uncertainties with the evidence base, followed by high-quality translation research if future studies suggest benefit without harmful effects on antibiotic resistance patterns.

### Future research

We found a very high level of agreement that further SDD research was ethical, and most participants would support their centre being involved in a RCT to evaluate the effectiveness of SDD. The potential of SDD to increase antibiotic resistance, despite being identified as a very important issue, would not necessarily limit trial participation. There were high levels of agreement that such a trial should include pre-trial, during-trial and post-trial monitoring of antibiotic resistance, however. Mortality was favoured as the primary outcome, with a cost–benefit analysis seen as desirable. It is clear with the strong weighting participants gave to antibiotic resistance that this outcome needs to be given equal stance in any future studies. Participants identified that the control arm should receive VAP bundles in the UK and Canada, and usual care in Australia and New Zealand. This could be influenced by the lack of national VAP guidelines in Australia or New Zealand. Further data on trial design issues were identified during contemporary interviews of intensive care triallists as another part of our research programme (JJ Francis, EM Duncan, ME Prior, GS MacLennan, SU Dombrowski, G Bellingan, MK Campbell, MP Eccles, L Rose, KM Rowan, R Shulman, APR Wilson, BH Cuthbertson, Selective decontamination of the digestive tract in critically ill patients treated in intensive care units: a mixed-methods feasibility study (the SuDDICU study), *Health Technology Assessment* 2013, in press). Such data can help inform practitioners and researchers on the need, appropriateness and design of future research.

### Strengths and limitations

A strength of this study was its grounding in a theoretical framework that enabled us to distinguish between factors related to the clinical evidence and potential barriers related to more practical issues to do with professional roles, resources and the management of change. Further strengths of this study were inclusion of four key clinical stakeholder groups with influence on SDD policy or delivery, thereby enabling a wide range of professional opinions from those likely to influence local policies. The study was completed by 118 clinicians from the four different groups from three geographical regions with high retention across rounds, making this the largest Delphi study to date in intensive care to our knowledge.

There was evidence of high stability of responses between Rounds 2 and 3, indicating that, when given three opportunities to consider their opinions, and viewing the spread of opinions across the cohort, participants reported consistent opinions about SDD over time. Indices of stability may mask individual instability if different stakeholder groups change their opinions in opposite directions. In this study there was evidence of stability at both the individual and group levels. This enhances our confidence that the identified opinions will be relevant over time, unless the profile of evidence, or the knowledge of the evidence, changes.

It is rare in Delphi studies to identify consensus around uncertainty (for example, in the range 4 to 6 on the nine-point scale), where this exists, and to contrast this with consensus around agreement (range of 7 to 9 on the nine-point scale). Given the overarching objective of the study, consensus around uncertainty was of great importance.

Self-rated knowledge of the field was generally high, although one-third of the participants rated their knowledge of the evidence base as uncertain or low. This variation in knowledge of the evidence base is a potential limitation of the study because it makes stated uncertainty difficult to interpret. There has been longstanding debate regarding the meaning of the neutral response in Likert scales [26]. This point is important because it could lead to an incorrect assessment of the presence of equipoise and the move to conduct a new clinical trial of SDD if neutral scores on key items were interpreted as equipoise or uncertainty when actually they could reflect lack of knowledge. By close scrutiny of the data we have identified that participants who rated their SDD knowledge as high were strongly represented among those who endorsed the neutral response relating to the consequences of SDD, reflecting clinical equipoise. We did not ask any questions about the participants’ views on the play off between mortality benefit and antibiotic resistance rates, but a separate publication reports on this point from the perspective of the ethical issues discussed by the participants.

Other weaknesses include the risk that our sampling framework failed to capture all important opinions on this subject. However, evidence from one randomised study indicates that the Framework can result in the generation of a wider range of opinions than using more open approaches to eliciting opinion [27]. Furthermore, in the Round 2 and Round 3 questionnaires we included all opinions emerging from the Round 1 interviews, including minority opinions, to ensure that all opinions were considered by all participants in the questionnaire rounds. The sample size was appropriate for a Delphi study but was numerically small when considering the scale of clinical practice across the four nations. Finally, the sample was taken from English-speaking countries only. Although these countries are widely geographically situated, we acknowledge that this is a limitation of our study.

## Conclusions

This theory-informed, international assessment of opinions about SDD has shown that further clinical research in this area needs to have significantly greater validity and generalisability. Clinicians do not currently plan to implement SDD into their practice without further supportive evidence. Future research needs to provide definitive answers on the clinical effectiveness of SDD, including in clinical environments with existing high antibiotic resistance rates, and on the effects of SDD on antibiotic resistance patterns (where endemic rates of antibiotic resistance are already high). It is clear from our study that any trial should include monitoring of antibiotic resistance before, during and after the trial. Mortality was favoured as the primary outcome and a control arm receiving VAP bundles or standard care was favoured. However, the topic was not seen currently as high priority and this may limit interest in future SDD trials. Any proposed future trials of SDD should take these factors into account.

## Key messages

• The evidence base for the effectiveness of SDD is strong but SDD will not be more widely implemented without further supportive evidence.

• Participants believe that further clinical research in this area needs to have significantly greater validity and generalisability.

• Further research must include monitoring of antibiotic resistance rates before, during and after the trial.

• SDD was not seen as a high priority and this could limit interest in further research.

## Abbreviations

HAI: Hospital-acquired infections; IQR: Interquartile ranges; RCT: Randomised controlled trial; SDD: Selective decontamination of the digestive tract; VAP: Ventilator-associated pneumonia.

## Competing interests

The authors declare that they have no competing interests.

## Authors’ contributions

BHC, MKC, GM, IMS, GB and JJF made substantial contributions to conception and design of the Delphi study, acquisition of data and analysis for the Delphi and interpretation of data using the study psychological framework. They drafted the article and revised it critically for important intellectual content. EMD, APM, ECW, MEP, LT, and LR made substantial contributions to the acquisition of data and analysis for the Delphi study and interpretation of data using the study psychological framework. They also helped draft the article and revised it critically for important intellectual content. All authors read and approved the final manuscript.

## Supplementary Material

Additional file 1: Table S2 Presenting all study questions and participants’ agreement and importance ratings. Click here for file

Additional file 2: Table S1 Presenting a breakdown of Delphi participation by stakeholder group. Click here for file

Additional file 3: Figure S1 Comparing key stakeholder groups for the statement ‘I am opposed to SDD’. Click here for file

Additional file 4: Figure S2 Comparing key stakeholder groups for the statement ‘The risks of SDD outweigh the benefits’. Click here for file

## The SuDDICU International Collaboration

SuDDICU UK study group: Jill Francis, international methods lead, Professor of Health Psychology, Health Psychology Group, Health Services Research Unit, University of Aberdeen, UK; Geoff Bellingan, UK clinical lead, Director, ICU, University College Hospital, London, UK; Marion Campbell, Director, Health Services Research Unit, University of Aberdeen, UK; Brian Cuthbertson, Canadian and International lead, Chief, Department of Critical Care Medicine, Sunnybrook Health Sciences Centre, Toronto, Ontario, Canada; Eilidh Duncan, Post-doctoral Research Fellow, Health Psychology Group, Health Services Research Unit, University of Aberdeen, UK; Martin Eccles, Professor of Clinical Effectiveness, University of Newcastle, UK; Graeme MacLennan, Senior Statistician, Health Services Research Unit, University of Aberdeen, UK; Maria Prior, Post-doctoral Research Fellow, Health Psychology Group, Health Services Research Unit, University of Aberdeen, UK; Louise Rose, Assistant Professor of Nursing, University of Toronto, Ontario, Canada; Kathy Rowan, Director and Professor, Intensive Care National Audit and Research Committee, London, UK; Rob Shulman, ICU Pharmacist, University College Hospital, London, UK; Peter Wilson, Medical Microbiologist, Department of Medical Microbiology, University College Hospital, London, UK.

SuDDICU Canada study group: Brian Cuthbertson, Canadian and International lead, Chief, Department of Critical Care Medicine, Sunnybrook Health Sciences Centre, Toronto, Ontario, Canada; Jill Francis, international methods lead, Professor of Health Psychology, Health Psychology Group, Health Services Research Unit, University of Aberdeen, UK; Karen Burns, Associate Professor, St Michaels Hospital, Toronto, Ontario, Canada; Deborah Cook, Canada Research Chair in Critical Care, McMaster University, Hamilton, Ontario, Canada; Peter Dodek, Professor, Center for Health Evaluation and Outcome Sciences, St. Paul’s Hospital, Vancouver, British Columbia, Canada; Niall Ferguson, Associate Professor, University Health Network, Toronto, Ontario, Canada; Jeremy Grimshaw, Professor, Ottawa Health Research Institute, University of Ottawa, Canada; Richard Hall, Professor of Anesthesiology, Pharmacology and Medicine, Associate Professor of Surgery, Dalhousie University and The Queen Elizabeth II Health Sciences Centre, Halifax, Nova Scotia, Canada; Lynn Johnston, Professor of Medicine, Dalhousie University, Halifax, Nova Scotia, Canada; Salmaan Kanji, Clinical Pharmacy Specialist, Critical Care Unit, The Ottawa Hospital, Ottawa, Ontario, Canada; John Marshall, Professor of Surgery, St Michaels Hospital, Toronto, Ontario, Canada; Lauralyn McIntyre, Associate Professor, Clinical Epidemiology, Ottawa Health Research Institute, Ottawa, Canada; John Muscedere, Associate Professor of Medicine, Intensivist Kingston General Hospital, Kingston, Ontario, Canada; Joe Pagliarello, University of Ottawa, Ontario, Canada; Louise Rose, Assistant Professor of Nursing, University of Toronto, Ontario, Canada; Laura Todd, Department of Primary Care, University of Toronto, Ontario, Canada; Fiona Webster, Education Scientist, Department of Family and Community Medicine, University of Toronto, Ontario, Canada; Charles Weijer, J.L. Rotman Institute of Science and Values, Western University, London, Ontario, Canada; Elisabeth Wells, Research Associate, Centre for the Study of Social and Legal Responses to Violence, University of Guelph, Ontario, Canada.

SuDDICU Australia and New Zealand Study Group: Ian Seppelt, Australia/New Zealand lead, Sydney Medical School – Nepean, University of Sydney, and the George Institute for Global Health, Sydney, New South Wales, Australia; Brian Cuthbertson, Canadian and international lead, Chief, Department of Critical Care Medicine, Sunnybrook Health Sciences Centre, Toronto, Ontario, Canada; Louise Cole, Nepean Hospital, Penrith, New South Wales, Australia; Arina Dan, The George Institute for Global Health, Sydney, Australia; Simon Finfer, Professor of Intensive Care, Royal North Shore Hospital and the George Institute for Global Health, Sydney, Australia; Jill Francis, international methods lead, Professor of Health Psychology, Health Services Research Unit, University of Aberdeen, UK; Parissa Glass, The George Institute for Global Health, Sydney, Australia; Jeff Lipman, Professor and Head of Intensive Care, Royal Brisbane and Women’s Hospital, Brisbane, Australia; Andrea Marshall, Professor of Acute and Complex Care Nursing, Gold Coast Hospital and Griffith University, Queensland, Australia; John Myburgh, Professor, Intensive Care, St George Hospital Sydney and the George Institute for Global Health, Sydney, Australia; David Paterson, Professor Infectious Diseases and Microbiology, Royal Brisbane and Women’s Hospital, Brisbane, Australia; Leonie Weisbrodt, Nepean Hospital, Penrith, New South Wales, Australia.
