# Supplementary material for: Clinical stakeholders’ opinions on the use of selective decontamination of the digestive tract in critically ill patients in intensive care units: an international Delphi study
Source: Crit Care. 2013 Nov 8;17(6):R266. doi: 10.1186/cc13096 (PMC4056354; doi:10.1186/cc13096)
Supplement: Additional file 1: Table S2 — Presenting all study questions and participants’ agreement and importance ratings. [file cc13096-S1.docx]

**Additional table 1-** Breakdown of Delphi participation by stakeholder group

| **Nation** | **Participant group** | **Delphi round** | | | |
| --- | --- | --- | --- | --- | --- |
|  |  | Round 1 | Round 2 | Round 3 | **Retention** |
| UK | Total  ICU physician  ICU pharmacist  MM / ID  ICU leaders  SDD used in current practice | 47  12  11  11  13  5 from 2 centres | 44  11  11  11  11 | 42  11  10  10  11 | 89%  92%  91%  91%  85%  11% |
| Australia and New Zealand | Total  ICU physician  ICU pharmacist  MM / ID  ICU leader  SDD used in current practice | 45  12  10  10  13  0 | 41  12  9  9  5 | 37  11  8  8  10 | 76%  92%  80%  80%  77%  0% |
| Canada | Total  ICU physician  ICU pharmacist  MM / ID  ICU leaders  SDD used in current practice | 49  13  13  10  13  0 | 43  13  12  8  10 | 39  13  10  8  8 | 80%  100%  77%  80%  55%  0% |
| Total (percentage retention) |  | 141  (100%) | 128  (91%) | 118  (82%) | Overall retention  82% |

Abbreviations- ICU- intensive care unit, MM- clinical microbiologist specialist; ID- infectious disease specialist.
