# Supplementary material for: Clinical stakeholders’ opinions on the use of selective decontamination of the digestive tract in critically ill patients in intensive care units: an international Delphi study
Source: Crit Care. 2013 Nov 8;17(6):R266. doi: 10.1186/cc13096 (PMC4056354; doi:10.1186/cc13096)
Supplement: Additional file 2: Table S1 — Presenting a breakdown of Delphi participation by stakeholder group. [file cc13096-S2.docx]

**Additional table 2- All question and participants agreement and importance ratings summarised as medians, 25th**

**and 75^th^ centiles and categorised according to topic area of question.**

|  | **25^th^ centile** | **Median** | **75^th^ centile** | **Importance rating** |
| --- | --- | --- | --- | --- |
| **Opinions on the relevance of SDD** | | | | |
| I am opposed to SDD* | 2 | 5 | 5 | na |
| My hospital tries to reduce antibiotic use* | 7 | 8 | 9 | 8 (7-9) |
| I know the SDD evidence base well enough to have an informed opinion regarding its use | 4 | 6 | 7 | 8 (6-8) |
| We are addressing Hospital Acquired Infections using other strategies* | 8 | 8 | 9 | 7 (6.5-9) |
| We are addressing Ventilator Associated Pneumonia using other strategies* | 7 | 8 | 9 | 7 (7-8) |
| Our unit VAP rates are low | 5 | 7 | 8 | 7 (6-8) |
| I am opposed to the IV component of SDD | 5 | 6 | 7 | 7 (5-8) |
| SDD is not on my Unit’s list of clinical priorities* | 7 | 8 | 9 | 6 (5-8) |
| SDD is not a topic of discussion among my colleagues | 6 | 8 | 9 | 6 (5-7) |
| Prophylactic antibiotic use in SDD is at odds with my professional training | 5 | 6 | 7 | 5 (5-7) |
| Prophylactic antibiotic use in SDD is at odds with my professional responsibilities* | 3 | 4 | 6 | 5 (5-7) |
| **The opinions of key stakeholders of the internal / external validity and adequacy of the existing evidence base for SDD? (research question 1)** | | | | |
| Research to date has not adequately addressed concerns about antibiotic resistance and SDD | 6 | 7 | 8 | 8 (7-9) |
| The SDD evidence base in not generalisable to my country** | 5 | 6 | 7 | 7 (5-8) |
| The SDD evidence base has been generated in countries with different resistance profiles to my country | 5 | 7 | 8 | 7 (5-8) |
| The SDD evidence base is not generalisable to my patient population | 3 | 5 | 7 | 7 (5-8) |
| **The opinions of key stakeholders about the likely positive and negative consequences of implementing SDD in ICUs and what is the relative importance of these beliefs in influencing overall opinions about SDD? (Research question 2)** | | | | |
| SDD increases antibiotic resistance** | 5 | 6 | 7 | 8 (7-9) |
| SDD would increase ICU *Clostridium difficile* infections** | 5 | 5 | 6 | 8 (6-8) |
| Overall, SDD benefits the patients to whom it is delivered** | 5 | 6 | 7 | 7 (5-8) |
| The risks of SDD outweigh the benefits** | 4 | 5 | 5 | 7 (6-8) |
| There is no mortality benefit associated with SDD | 3 | 5 | 5 | 7 (6-8) |
| SDD reduces length of stay | 5 | 5 | 6 | 7 (5-8) |
| SDD reduces Hospital Acquired Infections** | 5 | 6 | 6 | 7 (6-8) |
| SDD reduces VAP | 5 | 6 | 7 | 7 (5-8) |
| Overall, SDD is cost effective | 5 | 5 | 5 | 7 (5-8) |
| **Opinions of key stakeholders about the likely barriers to implementing SDD in ICUs (research question 3)** | | | | |
| The decision to adopt SDD requires consensus between my colleagues | 8 | 9 | 9 | 8 (7-9) |
| The decision to adopt SDD requires a review and appraisal of the current best evidence | 8 | 9 | 9 | 8 (7-9) |
| Part of the decision to adopt SDD requires agreement about which patients will receive it | 8 | 8 | 9 | 8 (7-9) |
| There are no national guidelines about SDD | 7 | 8 | 9 | 7 (6-8) |
| The skills required to administer SDD fall within the competencies of our ICU nursing staff | 8 | 9 | 9 | 7 (6-8) |
| There are conflicting opinions on antibiotic use among Clinical Microbiologists / ID Physicians and ICU physicians | 6 | 7 | 8 | 7 (5-8) |
| SDD would be a dramatic shift from our current practice | 7 | 8 | 9 | 7 (5-8) |
| I know to which patients I would administer SDD | 3 | 5 | 7 | 7 (5-8) |
| SDD is straightforward to deliver | 5 | 6 | 8 | 6.5 (5-8) |
| SDD drugs are expensive | 3 | 5 | 6 | 6 (5-7) |
| SDD increases nursing workload | 6 | 7 | 7 | 6 (5-7) |
| SDD causes unpleasant side-effects for patients | 4 | 5 | 5 | 6 (5-7) |
| The use of pastes may interfere with other treatments | 4 | 5 | 6 | 6 (5-7) |
| I am reassured that our position on SDD adoption is in line with other hospitals | 5 | 6 | 7 | 5 (5-7) |
| I could influence whether SDD is adopted in my hospital | 7 | 7 | 8 | 5 (5-7) |
| SDD increases pharmacy workload | 6 | 6 | 7 | 5 (4-7) |
| Educating staff would be expensive | 3 | 4 | 5 | 5 (4.5-6) |
| **Opinions of key stakeholders on the feasibility of further SDD research and whether these professional groups are likely to participate? (research question 4)** | | | | |
| It is ethically acceptable to conduct further RCTs evaluating the effectiveness of SDD | 7 | 8 | 9 | na |
| My concerns about antibiotic resistance limit my willingness to participate in future RCTs of SDD | 3 | 5 | 7 | na |
| I would support my centre being involved in a study to promote the adoption of SDD | 5 | 7 | 8 | na |
| I would support my ICU participating in a nationwide randomised control trial (RCT) of SDD | 7 | 8 | 9 | na |
| I would be more likely to participate RCT if mortality is the primary end-point*** | 5 | 7 | 8 | na |
| I would be more likely to participate RCT if cost-benefit analysis was included*** | 7 | 8 | 9 | na |
| I would be more likely to participate RCT if it included pre-, during- and post-trial monitoring of antibiotic resistance in patients in the RCT*** | 8 | 9 | 9 | na |
| I would be more likely to participate RCT if it included pre-, during- and post-trial monitoring of antibiotic resistance in all patients whether in the RCT or not*** | 8 | 9 | 9 | na |
| I would be more likely to participate if patients in the control arm received VAP bundles as usual care (including chlorhexidine mouthwash / gel and head up positioning)***, # | 8 | 9 | 9 | na |
| I would be more likely to participate if patients in the control arm received usual care ## | 8 | 9 | 9 | na |

*Data graphically presented in figure 1, **Data graphically presented in figure 2,

***Data graphically presented in figure 3, ****Data graphically presented in figure 1, # UK and Canada data only; ##Australia/New Zealand question only. These questions were the only questions changed between nations and reflected published variations in standards of care between countries related to control group management.
