# Supplementary material for: Clinical stakeholders’ opinions on the use of selective decontamination of the digestive tract in critically ill patients in intensive care units: an international Delphi study
Source: Crit Care. 2013 Nov 8;17(6):R266. doi: 10.1186/cc13096 (PMC4056354; doi:10.1186/cc13096)

**Additional figure 1-** Figure comparing key stakeholder groups for the questions “I am opposed to SDD”.


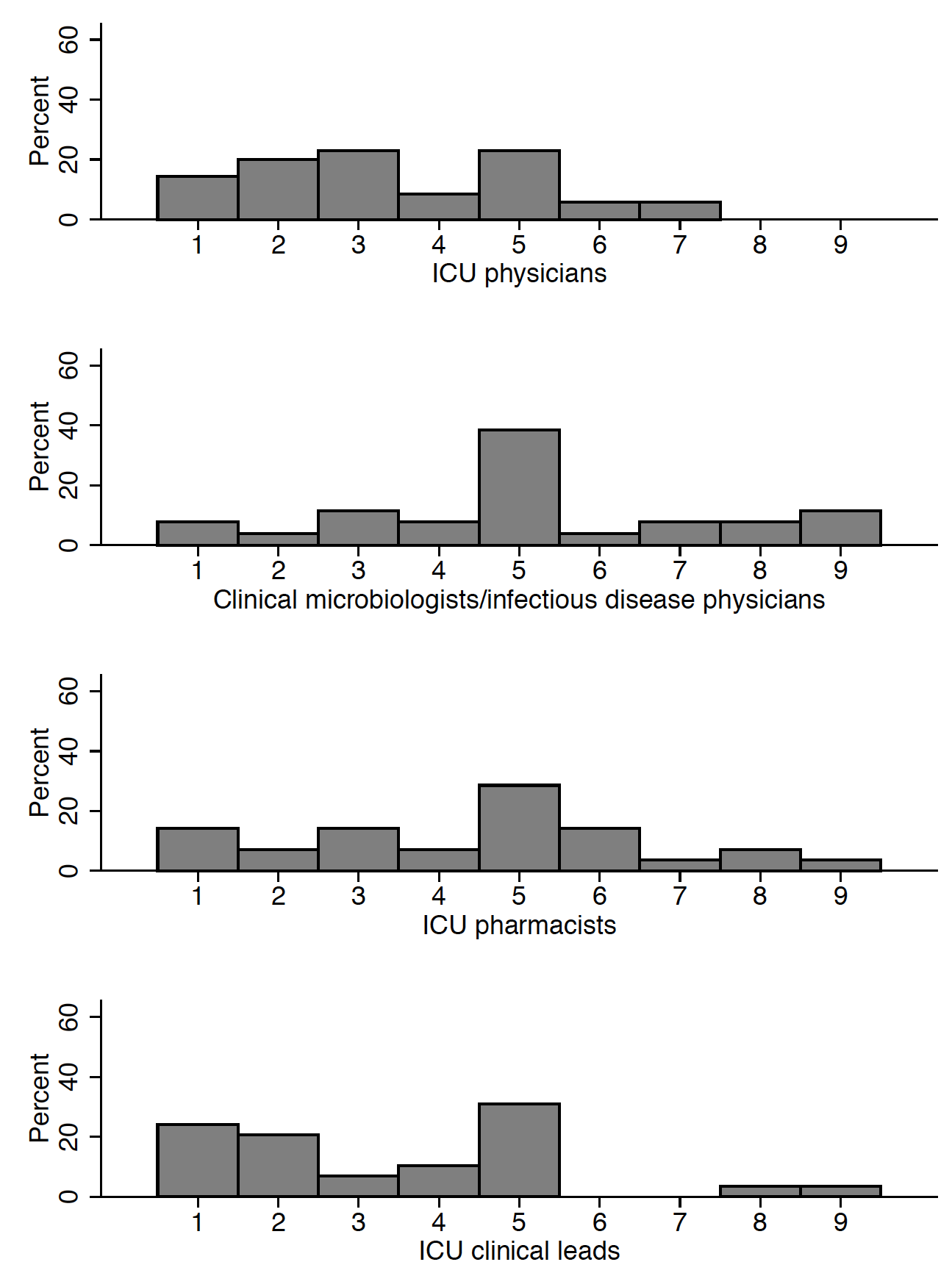

Supplement: Additional file 3: Figure S1 — Comparing key stakeholder groups for the statement ‘I am opposed to SDD’. [file cc13096-S3.docx]
